# Developing an advanced prediction model for new employee turnover intention utilizing machine learning techniques

**DOI:** 10.1038/s41598-023-50593-4

**Published:** 2024-01-12

**Authors:** Jungryeol Park, Yituo Feng, Seon-Phil Jeong

**Affiliations:** 1 Technology Policy Research Division, Electronics and Telecommunications Research Institute (ETRI), Daejeon, South Korea; 2 Management Information Systems, Chungbuk National University, Cheongju, South Korea; 3 Department of Computer Science, BNU-HKBU United International College, Zhuhai, Guangdong China

**Keywords:** Psychology, Human behaviour

## Abstract

In recent years, the turnover phenomenon of new college graduates has been intensifying. The turnover of new employees creates many difficulties for businesses as it is difficult to recover the costs spent on their hiring and training. Therefore, it is necessary to promptly identify and effectively manage new employees who are inclined to change jobs. So far previous studies related to turnover intention have contributed to understanding the turnover phenomenon of new employees by identifying factors influencing turnover intention. However, with these factors, there is a limitation that it has not been able to present how much it is possible to predict employees who are actually willing to change jobs. Therefore, this study proposes a method of developing a machine learning-based turnover intention prediction model to overcome the limitations of previous studies. In this study, data from the Korea Employment Information Service's Job Movement Path Survey for college graduates were used, and OLS regression analysis was performed to confirm the influence of predictors. And model learning and classification were performed using a logistic regression (LR), k-nearest neighbor (KNN), and extreme gradient boosting (XGB) classifier. A novel finding of this research is the diminished or reversed influence of certain traditional factors, such as workload importance and the relevance of one's major field, on turnover intention. Instead, job security emerged as the most significant predictor. The model's accuracy rates, highest with XGB at 78.5%, demonstrate the efficacy of applying machine learning in turnover intention prediction, marking a significant advancement over traditional econometric models. This study breaks new ground by integrating advanced predictive analytics into turnover intention research, offering a more nuanced understanding of the factors influencing the turnover intentions of new college graduates. The insights gained could guide organizations in effectively managing and retaining new talent, highlighting the need for a focus on job security and organizational satisfaction, and the shifting relevance of traditional factors like job preference.

## Introduction

Recently in Korea, the problem of youth employment, especially the deterioration of employment for college graduates, has become a social issue. However, companies are also complaining of a shortage of human resources, and one of the causes is the frequent turnover of new employees with college graduates^1^. According to the Ministry of Trade Industry and Energy, the early retirement rate of experienced people is 13%, while the early retirement rate of new employees is 66%^2^. According to a survey of economic activities by Statistics Korea, if young people aged 15–29 quit their first job, the average service period is 1.9 months. While most young people are eager to secure employment, a paradox emerges as many of those who successfully obtain jobs soon contemplate either changing their positions or resigning altogether^3^. We can look at the attrition of new hires and college graduates from two opposing perspectives. There is a view that new employee turnover is a job search process in the initial labor market entry process. There is a view that finding better working conditions is economical and rational^4^. However, failure to settle in the early stages of a career and the accumulation of unstable labor market experience can negatively affect an individual's career development, future wages and working conditions^5,6^. The turnover of these new employees can cause many difficulties for companies. This is because it is difficult to recover the costs spent on employee selection and training^7^. Also, turnover is contagious. New employees know much about the external labor market in the job search process, so they view the organization more objectively. Therefore, employees who see them change jobs also recognize a problem with the organization and tend to change jobs when they have the opportunity^8,9^. Therefore, quickly selecting and managing new employees with turnover intention can reduce the cost of hiring new employees and managing personnel.

However, it is not easy for companies to measure the turnover intention of new employees. Turnover intention is a sensitive issue for individuals, often concealed due to concerns about potential repercussions if disclosed externally^10^. Also, even if it disclosed, it is not easy to determine whether the content is true. Companies are facing difficulties in selecting and managing new employees with turnover intentions. Therefore, it is time to research ways to classify new employees with turnover intention quickly. Furthermore, prior studies^11–13^ have analyzed the causal relationship between dependent and independent variables using traditional econometric models but have limitations in that they have been unable to suggest whether new employees with actual turnover intentions can be predicted with these independent variables. In the case of rapid turnover among early career workers, predicting the dependent variable itself can be a significant problem^14,15^.

The turnover intention is being studied a lot in organizational psychology. Turnover intention refers to the degree of perception that people want to leave their current job^16^. Although they did not act, it indicates a tendency toward turnover. It can be said to be the degree to which an alternative to leaving the current organization and working in another organization is explored and considered. Previous studies on turnover intention identified various factors affecting turnover intention^1,17–20^. These research results helped improve the understanding of the turnover phenomenon. Turnover intention is the variable that has the most direct and robust influence on turnover^21^. According to Brown and Peterson, although an employee's turnover intention does not necessarily lead to turnover, turnover intention plays a role in effectively predicting the actual behavior of turnover^22^. This result supports the research result of Alderfer theory of reasoned action, which shows that the correlation between behavioral intention and actual behavior is very high^23^. Previous Studies confirmed that turnover intention and actual turnover had a high correlation^24–27^. As the such, turnover intention is highly correlated with turnover and is used as a dependent variable in place of turnover^24^. Therefore, the turnover intention is one of the important variables in the field of organizational psychology. The turnover intention in this study is, 'Are you engaged in activities to find another job?'. Looking at the turnover intention that contains actions from a practical point of view, it will be possible to understand the turnover phenomenon of new college graduates and a plan to manage them. Previous studies that identified the factors affecting this turnover intention are as follows.

A meta-analysis of previous studies on turnover intention revealed that factors influencing this phenomenon have been extensively studied^28^. These factors include personal characteristics such as age, years of service, gender, and educational status; job-related factors like salary, job performance, and overall job satisfaction; as well as external factors such as unemployment, new employment rate, and perception of employment.

It was found that reduced emotional and normative commitment were associated with increased turnover intention among new nurses^29^. Another study argued that perceptions of distributive justice, procedural justice, and interactional justice can reduce turnover intention in the context of organizational citizenship behavior^30^. Research identified job stress and sleep disorder as significant factors impacting turnover intention among new nurses during the 8th week of employment^31^. A study found that poor workplace behavior, high levels of work-related stress, and poor work-life balance were factors influencing turnover intention among new project management professionals in the construction field^32^. It was confirmed that citizenship behavior can mediate the relationship between organizational learning culture and turnover intention^33^. Another study identified subjective norms and training as major factors impacting turnover intention among new employees in the Hong Kong hotel industry^34^. Research found that the effectiveness of the onboarding program was an important factor influencing turnover intention for new employees with less than 2 years of experience in the ICT industry^35^. This study focuses on individual psychological changes as a factor influencing turnover intention. Specifically, it examines job selection motivation, satisfaction of needs, and suitability with the job as potential influences. It was found that job dissatisfaction under poor working conditions was related to job search, which was also related to actual turnover, using national representative data obtained from national registers^36^.

Motivation Plays a very important role in humanistic psychology, which presents human free will as a core concept^37,38^. Motivation was also defined motivation as a determinant that makes individuals put their efforts into and maintain specific tasks^39^. Motivation was defined as a belief that induces or sustains behavior so people can achieve their desired goals and achievements^40^. Another study defined motivation as a force that not only causes an action to occur but also suggests its direction, energizes it, and sustains it over time^41^. According to Self-Determination Theory (SDT), human behavior is caused by multiple motivations, and the outcome of the behavior depends on whether the motivation is intrinsic or extrinsic^37^. From this point of view, various studies have been conducted to determine what motivates people to choose a job. In general, the intrinsic motivation for choosing a job is an individual expectation, fulfillment, and satisfaction, and the extrinsic motivation for choosing a job is a material environment, human relationships, and rewards^42,43^. Therefore, the motivation for job selection is the degree to which the intrinsic and external motivation for the job work when an individual chooses a job.

The previous studies that suggested the factors of job choice motive are as follows. A study showed that working environment, opportunities to learn new things, future career prospects, challenging work, recognition from others, matching of aptitudes, job security, welfare benefits, working environment, ease of employment, working hours, promotion opportunity, time leeway, salary, bonus, ease of job transfer, self-actualization, and potential for self-development were suggested as motivating factors^44^. Another study suggested salary, employment stability, mental and physical relaxation, recognition from others, influence on others, voluntarily setting and achieving goals, work autonomy, and the possibility of acquiring new knowledge as motivation factors^45^. It was also suggested that motivation factors are motivation factors of income, employment stability, benefits, working hours, workload, working environment, company size, work distance, major suitability, aptitude and interest, job content, prospects, and social reputation^15^. This study conceptualizes human needs based on ERG theory, which categorizes and presents human needs from a general point of view in explaining job selection motivation^23^.

According to ERG theory, humans require existence (E), a need for relationship (R), and a need for growth (G), and the level of satisfaction of these needs affects motivation^23^. Individuals expect to satisfy their needs through their career choice^46^ and leave the organization if their needs are not satisfied through that career^47,48^. Therefore, the level at which the desire is satisfied can determine turnover intention. The three desires suggested by Alderfer (1972) are as follows.

**Existence needs (E)**: It is the need for humans to maintain survival. Organizations include salary, benefits, job security, and working environment.

**Relatedness needs (R)**: It is a desire to form and maintain relationships with groups (family, friends, and peer relationships, etc.) related to individuals. In organizations, include human relationships and desire for promotion.

**Growth needs (G)**: It is a desire to develop an individual and realize oneself. In the organization, including individual development potential, sense of achievement, aptitude and interest, and social recognition.

Previous studies to identify the factors of existence, relationship, and growth desire are as follows. A study presented his desire for existence: promotion, promotion evaluation fairness, salary satisfaction, relationship needs: recognition of peer work ability, job satisfaction, work environment, growth needs: personal development, confidence, and acquisition of new knowledge and skills^49^. Another study presented the desire for existence: financial compensation, employment stability, mental and physical relaxation, relationship needs: recognition from others, possibility of exerting influence on others, growth needs: goal setting and achievement, work autonomy, and new knowledge acquisition^45^. It also describes existence needs: security and stability for the future, financial compensation, promotion opportunities, welfare benefits, working hours, relationship needs: family atmosphere and support of the organization, smooth human relationships with colleagues, positive evaluation of superiors, Customers' positive evaluation, growth needs: suitability for job and individual, sense of achievement through work, acquisition of new knowledge and experience, opportunities for growth through work, social contribution of work, and sense of achievement through work are presented^50^. A study measured the ERG needs of knowledge-based employees using three variables: salary satisfaction, work relationship, and career growth^51^.

Personal-job fit can be seen as the level of agreement between the capabilities of individuals and the demands of their jobs^52^. This means the relationship between the characteristics of an individual and the characteristics of the job given to him^1^. Personal-job fit was related to job satisfaction, positive work attitude, quality of work performance, improved adaptability in new organizations, and reduced turnover intention^20,53,54^. Therefore, studies have investigated the relationship between various work-related attitudes and behaviors. A study presented a research model explaining individual-job suitability, defining it as two concepts: the degree to which the individual's ability and the ability required in the job match and the harmony between the individual's needs and the rewards provided in the job^55^. Looking at this, individual characteristics and job characteristics directly affect an individual's work-related attitude, the relationship between job characteristics and attitudes depends on the individual's ability and demand level, and the relationship between individual characteristics and attitudes depends on the job demand or supply level. A meta-analysis on 836 studies related to five individual-environmental suitability including individual-job suitability. Among them, 62 studies related to this analyzed in the case of individual-job suitability^56^. As a result, it was found to have a high correlation with job satisfaction, organizational commitment, and turnover intention. In addition, it found to be related to peer and boss satisfaction, organizational commitment, job performance, burden, tenure, turnover, organizational attraction, and recruitment intention. In this study, the subcategories of personal-job fit include level of work matching your level of education, skill level of job-matching your level of skill, and contents of work-matching your major.

As such, research to measure the turnover intention of college graduates is being conducted steadily. According to previous study, job-related factors, such as supervisor support, personnel systems, and job prospects, can increase turnover intention^57^. It was posited that the acquisition of competencies and understanding of organizational rules can reduce turnover intention^14^. It was also identified that workplace bullying, peer bullying, and supervisor bullying as factors that increase turnover intention^58^. Previous studies have primarily examined factors affecting turnover intention from the perspective of external factors, such as the job and organization. These findings aid in understanding the turnover phenomenon. However, considering that turnover intention is a tendency that represents individual psychological factors^16^, it is essential to focus on psychological changes such as motivation and the satisfaction of needs as the root cause of the problem. This is because motivation and desire are key research topics in explaining individual psychology, behavior, or organizational behavior^59^. From this perspective, this study differentiates itself from previous studies by focusing on the individual's psychological state and change by considering the motivation for job selection and satisfaction of needs of new employees and their suitability with the job.

Based on the above discussion, the following hypothesis was derived in this study.

### H1

As job preference are satisfied, turnover intention will decrease.

- As recognizing workload importance is satisfied, turnover intention will decrease.
- As recognizing the importance of the major field is satisfied, turnover intention will decrease.
- As recognizing work’s social reputation is satisfied, turnover intention will decrease.

### H2

As existence needs are satisfied, turnover intention will decrease.

- As satisfaction with wage or income is satisfied, turnover intention will decrease.
- As satisfaction with job security is satisfied, turnover intention will decrease.

### H3

As relatedness needs are satisfied, turnover intention will decrease.

- As satisfaction with organization is satisfied, turnover intention will decrease.
- As satisfaction with group membership is satisfied, turnover intention will decrease.

### H4

As growth needs are satisfied, turnover intention will decrease.

- As satisfaction with personnel system (promotion system) is satisfied, turnover intention will decrease.
- As satisfaction with individual development potential is satisfied, turnover intention will decrease.
- As satisfaction with work’s social reputation is satisfied, turnover intention will decrease.

And, the subcategories of personal-job fit include Level of work matching level of education, skill level of job matching level of skill, contents of work matching major. Based on the discussion above, it was judged that job choice motivation would affect turnover intention, and the following hypotheses were derived.

### H5

As personal-job fit increases, turnover intention will decrease.

- As the level of work matching the individual's level of education is met, turnover intention will decrease.
- As the skill level of the job aligns with the individual's skill level, turnover intention will decrease.
- As the content of work aligns with the individual's major, turnover intention will decrease.

Based on the above theoretical basis and hypothesis, this study aims to identify factors that influence turnover intention, and to establish and verify a predictive model. If a predictive model is built using public data and machine learning algorithms that measure the turnover intention of new college graduates, and the independent variables used in model construction are measured on new employees and put into the model, their turnover intention can be predicted. This approach not only provides practical implications for promptly identifying new employees with turnover intentions in situations where it is challenging to easily obtain sensitive information such as turnover intention, but also supplements the limitations of traditional econometric models that solely focus on causal analysis.

The structure of this study is as follows. First, through OLS regression analysis, the influence of job choice motivation and sub-variables of personal-job fit category on turnover intention was analyzed. After that, a prediction model of turnover intention was developed and analyzed through logistic regression (LR), k-nearest neighbor (KNN), and extreme gradient boosting (XGB) machine learning techniques. Through this, the factors affecting turnover intention were examined, and a plan to predict the turnover intention of new college graduates was presented.

## Methods

### Research model

This study aims to analyze the factors affecting the turnover intention of new college graduates and to suggest a plan to predict the turnover intention of new employees through these factors. Many studies have overlooked the risk of omitted variable bias by focusing on specific factors that influence turnover intention and not including important explanatory variables. While the selection of explanatory variables should be based on theory, it is virtually impossible to consider all potential explanatory variables that may affect turnover intention with current theories and existing research results. Furthermore, many existing studies have used methods such as factor analysis to reduce the number of explanatory variables in order to preserve degrees of freedom due to relatively small sample sizes, which can obscure the interpretation of specific aspects of the influencing factors. In other words, even one factor influencing turnover intention may have multiple facets, but this is not sufficiently considered in previous studies.

Therefore, in this study, each specific questionnaire item was separated as one variable. Job choice motivation is divided into three questions related to job preference. And based on the ERG theory, it is assumed that it is divided into 2 questions for existence needs, 2 questions for related needs, and 3 questions for growth needs, and will reduce turnover intention. In addition, personal-job fit is divided into three items, and it is assumed that it will reduce turnover intention. Job preference includes recognizing workload importance, recognizing the importance of the major field, recognizing work’s social reputation. Existence needs include satisfaction with wage or income, satisfaction with job security. Relatedness needs include satisfaction with organization, satisfaction with group membership). Growth needs include satisfaction with personnel system (promotion system), satisfaction with individual development potential, satisfaction with work’s social reputation).

**Figure 1:**
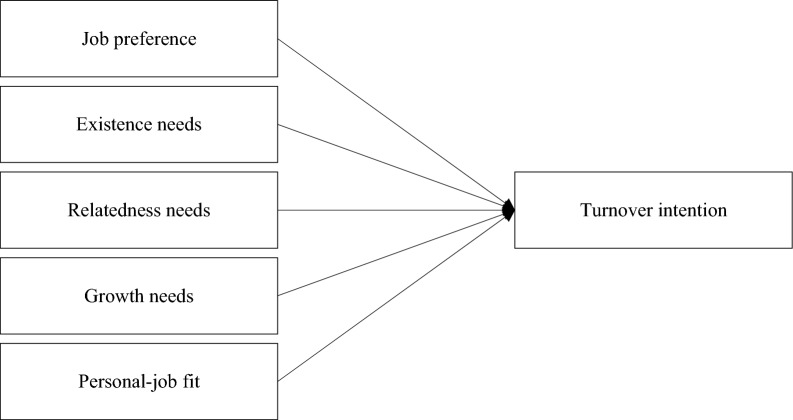
Research model.

In addition, to anticipate the anxiety of some researchers about the possibility of common method bias (CMB) problems in studies using one source, as in this study, procedural and statistical efforts were carried out^60–64^. Therefore, this study conducted a principal component analysis using scikit-learn's PCA class to perform Harman's single factor test. This analysis calculated the proportion of the first principal component in the total data variance. It was found that the first principal component accounted for 31% of the data variance. This percentage does not exceed the commonly used threshold of 50% for detecting significant common method bias in Harman's single factor test. Consequently, no significant common method bias was detected in this dataset. Figure 1 below is the model of our study, and Table 1 defines detailed variables. The satisfaction of group membership of a 7-point Likert scale, and all other variables were composed of a 5-point Likert scale.

**Table 1:** Influencing factors of turnover intention.

Constructs			Variables
Job choice motivation	Extrinsic motivation	Job preference	Recognizing workload importance
			Recognizing the importance of the major field
			Recognizing work's social reputation
		Existence needs	Satisfaction with wage or income
			Satisfaction with job security
		Relatedness needs	Satisfaction with organization
			Satisfaction with group membership
	Intrinsic motivation	Growth needs	Satisfaction with personnel system (promotion system)
			Satisfaction with individual development potential
			Satisfaction with work’s social reputation
Personal-job fit			Level of work matching level of education
			Skill level of job matching level of skill
			contents of work matching major

### Data

**Figure 2:**
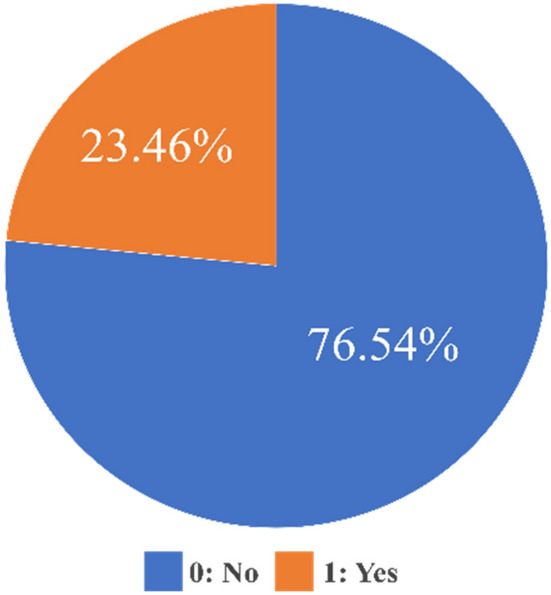
Turnover intention rate.

This study utilized data from the 2019 Graduates Occupation Mobility Survey (GOMS) conducted by the Korea employment information service, comprising 18,163 samples. The survey targets individuals who have graduated within the past two years and is recognized as government-approved statistics, officially accredited by the national statistical office (Statistics office approval number: 327004). Informed consent was obtained from all participants and their legal guardians, adhering to the relevant guidelines and regulations. Each year, GOMS establishes a panel of 18,000–20,000 people, or about 4% of them, with the population of graduates who completed a 2- to 3-year university or higher curriculum in the previous year. Therefore, it does not represent the Republic of Korea. However, the existence value of data is very high as it is used in the manpower supply and demand outlook in Korea. For the purpose of predicting turnover intention among currently employed respondents, 12,202 samples were selected, representing those who reported working in the past four weeks. The data consists of 6947 (56.9%) male and 5255 (43.1%) female. The study's dependent variable was turnover intention, assessed through the question, 'Are you ready to change your current job?' This was measured as a discrete variable with responses categorized as 'no (0)' or 'yes (1)'. The distribution of turnover intention was 9340 respondents (76.54%) indicating 'no' and 2862 (23.46%) indicating 'yes' (Fig. 2).

The Cronbach's Alpha value for the variables used in this study was 0.807 or higher. Discriminant validity was assessed by determining whether the smallest AVE squared value exceeded the largest correlation coefficient among the constituent concepts. As a result of the analysis, it was found that the criteria requirements^65^ for reliability and validity were met. Therefore, the reliability and validity of the measurement tools employed in this study were confirmed to be without any abnormalities.

### Influencing factors of turnover intention

This study analyzed path coefficient values and significance levels using ‘Ordinary Least Squares (OLS)’ regression analysis included in python's stats models package to examine the effects of 13 independent variables presented in Table 1 on turnover intention. OLS is a method to obtain a weight vector that minimizes the Residual Sum of Squares (RSS). This study presents average values, path coefficient values, t values, p values, and R^2^ values by referring to the indicators of previous studies using OLS regression analysis^66–68^.

### Machine learning techniques

Contrary to multiple linear regression, machine learning methods in artificial intelligence (AI) are increasingly used for prediction-related studies^69–76^. Machine learning is a way of implementing artificial intelligence, where computers discover new rules and patterns or make predictions about new data through data learning using algorithms^77^. Such machine learning can be divided mainly into supervised learning and unsupervised learning. Supervised learning involves labeling data and using it to predict future outcomes. It can be divided into regression and classification according to the characteristics of the results. Unsupervised learning is finding hidden patterns or structures in data by giving data to a computer but without a label. There is clustering and principal components analysis. In this study, classification techniques were used during supervised learning to classify given data based on discrete expectations for the digital divide. The classification technique is to build a model to distinguish different data dimensions according to specific criteria and predict discrete results for new data^78^.

The advantage of machine learning is the ability to use both categorical and numerical predictors to generate models by assessing linear and non-linear relationships between variables and the importance of each predictor. In regression analysis, a traditional statistical method, when many variables are used simultaneously, the basic assumptions about independent variables, such as exogeneity and homoscedasticity, are difficult to maintain, and the high correlation between variables can cause multicollinearity^9^. In contrast, the analysis of the accuracy of the machine learning-based prediction model assumes that dependent and independent variables are associated with each other. In addition, the roles that dependent variables play in predicting independent variables are analyzed, so the predictive power is unaffected even when multicollinearity is caused^79^. Therefore, an analysis can be performed even in the presence of many variables. These machine learning classification algorithms include LR, KNN, XGB.

LR is an analysis technique to prove the causal relationship between the independent and dependent variables. Here, the form of the dependent variable is categorical data, and when there are two categories, it's a binary logistic regression, and when there are more than two, it's a multinomial logistic regression.

1 $$In\left(\frac{\pi }{1-\pi }\right)=\upbeta 0+\upbeta 1{\text{X}}1+\upbeta 2{\text{X}}2+\cdot \cdot \cdot +\mathrm{\beta pXp}$$

KNN is a non-parametric method that, given some data, looks at the surrounding (neighbor) data and classifies it into a category that contains more data^80^. It is used in a state where the type of class to be classified is known, but the probability density function for each sample is not known. Since it is a labor-intensive method when large amounts of training data are given, it did not gain a reputation until the 1960s, but has been widely used in pattern recognition since computer performance improved. KNN generally calculates the distance between data and data through Euclidean distance. The formula is as follows.

2 $$f\left( {{\text{A}},{\text{B}}} \right) = \,\,\left| {{\text{x}}1 - {\text{x}}2} \right| + \left| {{\text{y}}1 - {\text{y}}2} \right|$$

XGB, a model developed by improving the boosting method of the decision tree, has an internal function of regularizing overfitting and carries out internal cross-validation at each trial^81^. Due to its excellent classification performance, XGB is often used in competitions, such as kaggle. Above all, the greatest advantage of XGB is its high practical usefulness. XGB allows for the derivation of important indices, which indicate relatively more important variables among various independent variables, so that the relative predictive power of various independent variables can be reviewed. Therefore, XGB was used in this study.

3 $${\mathcal{L}}^{\left(t\right)}={\sum }_{i=1}^{n} {l} (\mathcal{Y}_{i},{\widehat{\mathcal{Y}}_{i}}^{\left(t-1\right)}+{f}_{t}({x}_{{i}}))+\Omega ({f}_{t})$$

Various studies have used these machine learning techniques, such as credit card fraud detection, student satisfaction prediction, cyberbullying detection model construction, and youth suicide risk prediction. A study developed a predictive model for credit card fraud detection using public data on credit card transaction records and machine learning algorithms (LR, Naïve Bayes, KNN)^82^. The analysis results prove that KNN has the highest prediction accuracy. Another study developed a youth suicide risk prediction model using public data from the Korean adolescent risk behavior survey and machine learning algorithms (LR, RF, SVM, ANN, XGB)^83^. XGB showed the highest prediction accuracy. In previous study, a cyberbullying detection model was developed using twitter data and machine learning algorithms (Naïve bayes, KNN, DT, RF, SVM), and SVM showed the highest prediction accuracy^84^. Another study developed a student satisfaction prediction model using traditional regression analysis and machine learning algorithms (KNN, SVM, Light GBM, RF, ENet), and ENet showed the highest prediction accuracy^85^. Recently a study have highlighted the frequent use of machine learning techniques for data mining, including LR, SVM, DT, and ANN. Therefore, using machine learning algorithms to solve the digital divide can be a future exploratory direction^86^.

### Prediction model training

In this study, to predict turnover intention, using a supervised learning method during machine learning, data were trained on a prediction model, and then the accuracy was analyzed. The specific method is as follows. First, a turnover intention prediction model was constructed in which all the variables presented in Table 1 were set as independent variables, and turnover intention was set as the dependent variable. Second, the data was divided into 70% training set and 30% test set, and the model was trained with the training set, and then the prediction accuracy of the model analyzed with the test set. Third, the prediction accuracy of the prediction model was analyzed using LR, KNN, and XGB.

### Accuracy analysis

Meanwhile, cross-validation was performed four times to prevent the model from overfitting only a specific data set and to generalize the analysis results. On the other hand, the widely used accuracy, precision, recall, and f1-score are used as the performance evaluation indicators of the predictive model. The meaning and formula of each indicator are as follows.

*Accuracy* Accuracy is the most intuitive performance measure and is simply a ratio of correctly predicted observations to the total observations.

4 $$\left({\text{Accuracy}}\right)= \frac{TP+TN}{TP+FN+FP+TN}$$

*Precision*: Precision is the ratio of correctly predicted positive observations to the total predicted positive observations.

5 $$\left({\text{Precision}}\right)= \frac{TP}{TP+FP}$$

*Recall*: Recall is the ratio of correctly predicted positive observations to all observations in the actual “yes” class.

6 $$\left({\text{Recall}}\right)=\frac{TP}{TP+FN}$$

*F1-score*: F1-score is the weighted average of precision and recall.

7 $$\left({\text{F}}1-{\text{score}}\right)=2 \times \frac{1}{\frac{1}{Precision} + \frac{1}{Recall}} =2 \times \frac{Precision \times Recall }{Precision+Recall}$$

In order to obtain the above performance measures, a confusion matrix is required. Confusion matrix is also expressed as a 'mixed matrix', 'contemporaneous table', and 'error matrix'. Excluding the title of each row and column, it is an array composed of 2 × 2, and is expressed as follows. TP stands for True positive, which is actually True, when the prediction is judged to be True in the classification model. TN stands for True negative, which is actual False, when the prediction is judged to be False in the classification model. FP stands for False positive, which is actually False, which is when the prediction is judged to be true in the classification model. FN stands for False negative, which is actually True, when the prediction is judged to be False in the classification model.

## Results

### Analysis of influencing factors of turnover intention

As a result of analyzing the effect of the independent variables in this study on turnover intention through regression analysis, the three variables of job preference (recognizing workload importance, recognizing the importance of the major field, and recognizing work's social reputation) and one of growth needs All variables except for the variable of satisfaction with work's social reputation were found to have an effect (p < 0.001). To summarize this, Table 2 is as follows.

**Table 2:** Analysis result of influencing factors of turnover intention.

No	Variables	Mean	Coefficient	t-value	p >|t|	Hypothesis
Q1	Recognizing workload importance	3.955	0.056	1.319	0.187	Not supported
Q2	Recognizing the importance of the major field	3.708	0.050	1.412	0.158	Not supported
Q3	Recognizing work's social reputation	3.636	0.025	0.644	0.520	Not supported
Q4	Satisfaction with wage or income	3.291	− 0.113	2.521	0.012	Supported
Q5	Satisfaction with job security	3.786	− 0.252	5.675	0.000	Supported
Q6	Satisfaction with organization	3.612	− 0.928	15.389	0.000	Supported
Q7	Satisfaction with group membership	5.220	− 0.065	2.030	0.042	Supported
Q8	Satisfaction with personnel system (promotion system)	3.293	− 0.277	5.667	0.000	Supported
Q9	Satisfaction with individual development potential	3.616	− 0.377	7.840	0.000	Supported
Q10	Satisfaction with work’s social reputation	3.693	0.298	5.765	0.000	Not supported
Q11	Level of work matching level of education	2.984	− 0.255	3.251	0.001	Supported
Q12	Skill level of job matching level of skill	3.010	− 0.225	2.879	0.004	Supported
Q13	contents of work matching major	3.245	− 0.168	5.402	0.000	Supported

R^2^ 0.800.

### Analysis result of prediction accuracy of turnover intention

As a result of the turnover intention prediction model analysis, looking at the average values of the four sets of cross validation, accuracy showed the highest accuracy of XGB (0.785), followed by LR (0.783), and KNN (0.761). Precision showed that XGB showed the highest accuracy (0.806), followed by LR (0.798) and KNN (0.798). Recall showed that LR showed the highest accuracy (0.956), followed by XGB (0.942), and KNN (0.915). The f1-score showed that LR showed the highest accuracy (0.870), followed by XGB (0.869), and KNN (0.853). This is summarized as shown in Table 3.

**Table 3:** Analysis result of prediction models.

Set	Classifications	Accuracy	Precision	Recall	F1-score
Set 1	LR	0.788	0.806	0.951	0.873
	KNN	0.769	0.808	0.915	0.858
	XGB	0.785	0.811	0.936	0.869
Set 2	LR	0.770	0.782	0.958	0.861
	KNN	0.748	0.779	0.921	0.844
	XGB	0.772	0.789	0.945	0.860
Set 3	LR	0.800	0.817	0.957	0.881
	KNN	0.778	0.819	0.917	0.865
	XGB	0.802	0.828	0.940	0.880
Set 4	LR	0.776	0.787	0.958	0.864
	KNN	0.752	0.789	0.916	0.848
	XGB	0.781	0.796	0.946	0.865
Cross validation average	LR	0.783	0.798	0.956	0.870
	KNN	0.761	0.798	0.915	0.853
	XGB	0.785	0.806	0.942	0.869

*LR* logistic regression, *KNN* K-nearest neighbor classifier, *XGB* eXtreme gradient boosting.

### Feature importance

**Figure 3:**
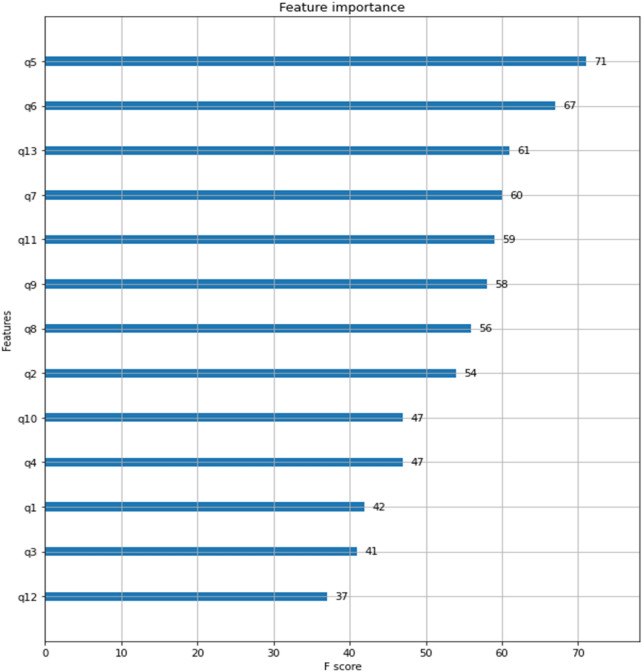
Feature importance results.

One of the advantages of XGB used in this study is that it can calculate the importance of independent variables input to the prediction model. This importance does not indicate the direction of turnover intention, but provides insight into the ranking of important variables in predicting turnover intention. In this study, the importance ranking of independent variables was analyzed through XGB's 'plot_importance' library (Fig. 3). The f1-score is an indicator of how often the corresponding feature was used when splitting a tree, and the higher the F score, the more often it is used for splitting a tree^87^. As a result of the analysis, the most important variable in predicting turnover intention was satisfaction with job security. After that, satisfaction with organization, and contents of work-matching your major appeared in order.

## Discussion

This study tried to analyze the factors affecting the turnover intention of new employees with college graduates while the turnover of new employees is intensifying and to suggest a way to predict new employees with turnover intention through these factors. In this study, 13 variables that affect turnover intention were verified. The result is existence needs (satisfaction with wage or income, satisfaction with job security), relatedness needs (satisfaction with organization, satisfaction with group membership), growth needs (satisfaction with personnel system (promotion system), satisfaction with individual development potential, satisfaction with Work's social reputation) and personal-job fit (level of work matching level of education, skill level of job matching level of skill, contents of work matching major) were found to have an effect on turnover intention.

Next, the results of the accuracy analysis of turnover intention prediction through 13 variables were examined based on the average value of each performance evaluation index. Accuracy showed the highest, with XGB showing 78.5%, but it did not show a significant difference with 78.3% of LR that followed, and KNN showed the lowest accuracy with 76.1%. Precision was highest with XGB at 80.6%, while LR and KNN showed 79.8% accuracy. On the other hand, in recall, LR showed the highest accuracy with 95.6%, followed by XGB with 94.2% and KNN with 91.5% accuracy. In f1-score, LR showed the highest at 87%, but there was no significant difference from 86.9% of XGB that followed, and KNN showed the lowest accuracy with 85.3%.

Upon analyzing the importance of variables, it was found that satisfaction with job security of existence needs was the most significant predictor of turnover intention. Additionally, it was found that satisfaction with the organization of relatedness needs, the alignment of work with one's major in terms of personal-job fit, and satisfaction with potential for individual development of growth needs were also of high importance.

The findings of this study are consistent with the results of previous research^88,89^, which have found that factors such as wage or income can reduce turnover intention. Specifically, job security, which is defined as the psychological state of feeling that one's job and employment status are secure^90^, has been identified as a factor that reduces job search behavior among new employees^91^, increases organizational commitment^92,93^. Thus, from the perspective of satisfying existence needs, it can be inferred that the satisfaction of wage or income and job security are factors that reduce turnover intention, which aligns with the results of previous studies.

Meanwhile, newcomers to society often face the challenge of adapting to new environments and forming human relationships^94^. If adaptation is not successful and satisfaction with relationships is low, individuals may experience continuous psychological stress and an increase in turnover intention^95,96^. Conflicts in relationships appear in terms of interpersonal relationships outside of work^97^, and it has been reported that when such conflicts occur in new employees who have not yet adapted to the organization, their intention to turnover increases^98–100^. This is believed to be related to psychological stress, as individuals may seek to leave the organization as a means of relieving negative emotions^101^. Additionally, organizational satisfaction and turnover intention have been identified as indicators of successful adaptation of new employees to the organization, and a high correlation has been reported between the two^102–104^. Furthermore, satisfaction with a team, a smaller concept than an organization, has also been found to influence turnover intention^56,105,106^. Therefore, from the perspective of satisfying relatedness needs, it can be inferred that an increase in satisfaction with the organization and group membership leads to a decrease in turnover intention. For individuals who are new to society, such as recent college graduates, personal growth is of paramount importance. Those in the early stages of the life-career development cycle should focus on exploring their career options and developing the skills necessary to excel in their chosen field. New employees, in particular, have a strong desire for self-realization through their work and tend to continually evaluate whether the organization they are working for can support their growth^1,107^. Additionally, they tend to view the organization's personnel system more objectively as they have not been in the workforce for very long^9^. As a result, it can be inferred that satisfaction with personnel system, satisfaction with individual development potential from the perspective of satisfying individual growth needs, can lead to a decrease in turnover intention, which is consistent with the findings of previous studies.

Alignment between an individual and their environment is known to lead to higher levels of performance, satisfaction, and reduced stress^108^. This means that when an individual's competencies align with the competencies required by the job, their job performance will be more positive^53,109^. As a result, personal-job fit has been identified as a factor that can increase organizational members' commitment and satisfaction and decrease turnover^48,52,110–112^. This can be explained by the attraction-selection-attrition theory advocated^47^. This is a series of processes in which people who are attracted to a specific organization or job apply, the organization selects more suitable people among them, and after the selection, those who fail to adapt to the organization and job and are not suitable are kicked out. Therefore, the subjects for whom the concept of suitability is important are mainly early entrants such as new employees^113^. Specifically, it has been reported that when there is a low alignment between an individual's education and their actual job, turnover intention increases^55^. Therefore, it can be inferred that the higher the person-job fit, the lower the turnover intention, which is consistent with the findings of previous studies.

However, our study found that job preference, specifically recognizing workload importance, recognizing the importance of the major field, and recognizing work’s social reputation of the work, is no longer a factor influencing turnover intention. These results differ from previous studies^114–116^, suggesting an information asymmetry between job suppliers and consumers. For example, previous research has found that workload is a significant factor influencing turnover intention among new employees^117^. However, our study found that recognizing the importance of workload before employment did not affect turnover intention. This may be due to the structural limitations of the Korean recruitment process, which only provides fragmentary information about jobs, making it difficult for prospective employees to accurately determine the workload. This could be estimated by looking at job postings on social media or internet sites. As a result, it is interpreted that the recognizing workload importance before employment did not affect turnover intention.

In addition, previous research has found a relationship between an individual's major and job prior to employment as a factor that reduces turnover intention^116^. However, this study found that the recognizing the importance of the major field did not have an impact on turnover intention. This is likely due to changes in the labor market and educational environment in Korea. Currently, due to the deterioration of the job market in Korea, there is a trend towards seeking employment regardless of one's major^118^. Furthermore, Korea has implemented a blind system in which personal information, such as name, age, school, and major, cannot be disclosed during recruitment. As a result, companies are unable to determine an applicant's major. In this context, companies are increasingly seeking versatile candidates, regardless of their majors, while universities are responding by offering convergence departments and majors. Therefore, it is believed that the results of this study, which showed that the relationship between the job prior to employment and the individual's major did not affect turnover intention are reflection of these changes. However, the study also found that contents of work-matching your major reduces turnover intention. In other words, even if the relevance to the major prior to employment does not affect turnover intention, if the compatibility with the major after employment is low, turnover will be considered. These findings highlight the importance of how majors in college can impact turnover intentions.

In addition, it has been reported that new employees value the reputation of their job or the organization in which they work^119,120^. A positive reputation for a job is known to increase satisfaction with the organization^121^ and reduce turnover intention^114,115^. However, according to this study, recognizing work's social reputation for pre-employment jobs does not affect turnover intention. However, a more important finding is that the greater the satisfaction with the social reputation of one's job after employment, the more likely they are to consider turnover. According to Social Identity Theory, individuals derive pride from their work through the social evaluation of others^122^. In particular, comparison between the current organization and other organizations strengthens self-esteem, allowing individuals to vicariously experience the organization's status and reputation, and to remain or become immersed in the organization^19^. In other words, it can be seen that a positive social reputation for work increases individual self-esteem and induces movement to a better place through organizational comparison.

Next, according to the results of this study, the highest accuracy was obtained when XGB was used. Recently, XGB claimed to perform relatively better than other classifiers in various competitions, such as kaggle^80^. Although the ensemble technique is superior in classification problem-solving, the results of this study did not show much difference from LR. As with public data, there are various variables, such as continuous and categorical variables. When the data size is different, it is not easy to guarantee which classification algorithm is the best for creating and analyzing a predictive model. The best method is to use various algorithms. It is a desirable method to apply and select the algorithm with the best performance among them. But, the XGB model is praised for its ability to rank the importance of features, but it is crucial to note that this does not indicate the (causal) direction of the relationship. Identifying that a variable is essential for predicting turnover is useful, but without understanding how it influences turnover (positively or negatively), the empirical estimates have limited practical applications.

Meanwhile, from the perspective of important variables in predicting turnover intention, this study results can be interpreted as having the needs strength effect, as proposed by ERG theory. According to this theory, individuals experience needs in the order of existence, relatedness, and growth, and as lower-level needs are met, the intensity of higher-level needs increases. Therefore, it is crucial to consider satisfaction with job security of existence needs, satisfaction with the organization of relatedness needs, and satisfaction with potential for individual development of growth needs when predicting turnover intention. However, the theory also suggests that the need strength effect can occur in the reverse direction, where even if higher-level needs are met, the intensity of lower-level needs may increase at any time. This should be taken into consideration, especially for early career individuals such as new college graduates, as their needs may change frequently.

This study advances the field of turnover intention research by integrating machine learning techniques with traditional econometric analysis. Key findings include the diminished or even inverse impact of certain variables previously thought to affect turnover intention, and the establishment of a hierarchy of variables critical in predicting turnover intention. A notable discovery is that low pre-employment recognition of a job's major field and social reputation can lead to increased turnover intention if post-employment desires remain unfulfilled. Additionally, this study highlights job security satisfaction as the most vital predictor of turnover intention among new college graduates, offering a more nuanced understanding of their turnover motivations. Practical applications of this study involve the development of a predictive model for turnover intention, using the methodology and variables outlined. It can help organizations identify and manage new employees at higher risk of turnover, potentially reducing related costs. The study’s comprehensive analysis of factors influencing turnover intention, such as existence, relatedness, and growth needs, along with personal-job fit, provides organizations with actionable insights. For instance, enhancing compensation, job security, positive organizational culture, career development opportunities, and aligning job roles with individual skills and education can effectively reduce turnover intention. Overall, this study not only expands the methodology for predicting turnover intention by applying machine learning but also underscores the importance of examining job choice motivation, individual need satisfaction, and job suitability to mitigate turnover intention in new employees. This offers valuable guidance for organizations in managing and retaining new talent.

The study has limitations, including its reliance on cross-sectional public data, making causality inference cautious. The findings, specific to university graduate newcomers, require further validation across diverse populations. Additionally, the data imbalance in turnover intention and the limited use of classification algorithms in the study suggest areas for future research enhancement. In addition, in future studies, it is believed that more diverse implications can be obtained by reflecting the characteristics of Generation Z, which refers to the younger generation born between the mid-1990s and the early 2000s, in the model. In particular, job security, organizational satisfaction, and work matching one’s major field were the most important variables in classifying employees with intention to turnover through XGB. In future studies, it is expected that more diverse practical implications can be derived if research focuses on these variables.

## Data Availability

The datasets analysed during the current study are available in the [Github] repository, [https://github.com/jrpark16/GOMS.git].
